# Time-Kill Kinetics of Rezafungin (CD101) in Vagina-Simulative Medium for Fluconazole-Susceptible and Fluconazole-Resistant* Candida albicans* and Non-*albicans Candida* Species

**DOI:** 10.1155/2018/7040498

**Published:** 2018-02-22

**Authors:** Jeffrey B. Locke, Amanda L. Almaguer, Joanna L. Donatelli, Ken F. Bartizal

**Affiliations:** Cidara Therapeutics, Inc., San Diego, CA, USA

## Abstract

**Background:**

While echinocandins demonstrate excellent efficacy against* Candida* species in disseminated infections and demonstrate potent minimal inhibitory concentration (MIC) values under standard susceptibility testing conditions, investigation under conditions relevant to the vaginal environment was needed. We assessed the antifungal activity and time-kill kinetics of the novel echinocandin rezafungin (formerly CD101) under such conditions, against* Candida* species relevant to vulvovaginal candidiasis (VVC).

**Methods:**

Susceptibility testing of fluconazole-susceptible and fluconazole-resistant* C. albicans*,* C. glabrata*,* C. tropicalis*,* C. parapsilosis*, and* C. krusei *was performed in RPMI at pH 7.0 and in vagina-simulative medium (VSM) at pH 4.2 for topical rezafungin, terconazole, fluconazole, and amphotericin B. Time-kill kinetics were evaluated for rezafungin and terconazole at 2, 8, 32, and 128 *μ*g/ml over 72 hours.

**Results:**

Rezafungin MIC values were the same or 2-fold higher in VSM/pH 4.2 versus RPMI/pH 7.0. Some* C. albicans* terconazole MIC values were lower, but most were significantly higher in VSM than in RPMI. Rezafungin was fungicidal against 11/14 strains and near-fungicidal against the others. Terconazole (128 *μ*g/ml) was fungicidal against* C. krusei *and near-fungicidal against susceptible* C. parapsilosis* but fungistatic versus all other strains evaluated.

**Conclusion:**

Rezafungin retained anti-*Candida* activity and fungicidal activity under in vitro conditions relevant to VVC.

## 1. Introduction

Vulvovaginal candidiasis (VVC) affects most women at least once in their lifetime [1] and a smaller subset (6–9%) will experience recurrent disease (RVVC) [2, 3]. In the United States and Europe, VVC is predominantly caused by* Candida albicans *(~76–89%) although non-*albicans Candida* species comprise larger proportions in other geographical regions [4]. Some studies suggest that the abundance of non-*albicans Candida* has increased over time in VVC infections [5, 6], particularly in recurrent disease [5, 7, 8]. Azoles are the standard of care for treatment of VVC; however, increasing rates of azole resistance (in both* C. albicans* and non-*albicans Candida* species, such as* Candida glabrata*, which exhibits higher rates of azole resistance, and* Candida krusei,* which is intrinsically azole-resistant) threaten the efficacy of this class [9, 10]. Furthermore, there are concerns regarding risk of relapse, drug-drug interactions, and safety during pregnancy for fluconazole (FLU) [11–13]. There are currently no FDA-approved therapeutics for RVVC and no novel agents have been marketed for VVC in more than 20 years, despite poor outcomes and unmet needs with current therapeutics [14, 15].

Echinocandins have been used as standard of care therapy for invasive and bloodstream* Candida* infections given their excellent safety profile and fungicidal mechanism of action which contribute to their high levels of efficacy clinically [16]. However, limited chemical stability has prevented currently approved echinocandins from topical formulation for use in indications such as VVC. Rezafungin (RZF, formerly CD101) is a novel echinocandin with antifungal potency and spectrum similar to currently approved echinocandins (caspofungin, micafungin, and anidulafungin) [17–19] in addition to a remarkable chemical and metabolic stability that enables topical formulations. A prior study demonstrated that in vitro antifungal activity of RZF was largely retained under low pH in standard fungal susceptibility testing media against clinical VVC isolates [20]. Whether CD101 fungicidal killing kinetics observed at neutral pH are also retained in the vaginal environment is unknown.

The purpose of this study was to evaluate the in vitro fungicidal activity of RZF through analysis of time-kill kinetics for FLU-susceptible and FLU-resistant* Candida *species under conditions and at a pH relevant to the vaginal microenvironment, using the topical azole VVC therapeutic, terconazole (TER), as a comparator.

## 2. Materials and Methods

### 2.1. Strains and Culture Conditions

The fourteen* Candida *strains evaluated in this study included* C. albicans* ATCC 44858,* C. glabrata* ATCC 200918,* C. krusei* ATCC 6258 and 14243 (American Type Culture Collection, ATCC; Chantilly, VA),* C. glabrata* MMX 7070,* Candida parapsilosis* MMX 7370,* Candida tropicalis* MMX 7255 and 7525 (Micromyx, LLC; Kalamazoo, MI),* C. glabrata* CG01,* C. parapsilosis* CP01 and CP02,* Candida tropicalis* CT02 (Wayne State University; Detroit, MI),* C. albicans* DPL001 (Rutgers University; Newark, NJ), and* C. albicans* R357 (Eurofins Panlabs, Inc.; St. Charles, MO) (Table 1). For each of the five* Candida* species evaluated, one fluconazole-susceptible (FLU-S) and two fluconazole-resistant (FLU-R) isolates were chosen, as classified per Clinical and Laboratory Standards Institute (CLSI) interpretive criteria [21], with the exception of* C. krusei* which is intrinsically azole-resistant. Where possible, VVC clinical isolates were included. All strains were cultured aerobically at 35°C on Sabouraud dextrose agar (SDA) plates prior to use in susceptibility and time-kill assays.

### 2.2. Antifungal Agents

Stocks of RZF (Cidara Therapeutics) were prepared fresh in 100% dimethyl sulfoxide (DMSO; Sigma, cat. number 276855) prior to use. Comparator antifungals, FLU (Alpha Aesar, cat. number J62015), TER (Fluka, cat. number 32355), and amphotericin B (AMB; Sigma, cat. number A2411) were also prepared in 100% DMSO according to CLSI guidelines [21].

### 2.3. Antifungal Susceptibility Testing

Susceptibility testing of* Candida* strains was performed using RPMI 1640 broth (MP Biomedicals, cat. number 1060124) that was buffered with 0.165 M 3-(*N*-morpholino) propanesulfonic acid (MOPS) and then adjusted to pH 7.0 with 1 N NaOH or in vagina-simulative medium (VSM) [22]. VSM was prepared with 3.5 g of NaCl/l, 1.4 g of KOH/l, 0.22 g of Ca(OH)_2_/l, 18 mg of bovine serum albumin/l, 2.2 g of 90% lactic acid/l, 1 g of glacial acetic acid/l, 0.32 g of 50% glycerol/l, 0.4 g of urea/l, 5 g of glucose/l, and 6.7 g of yeast nitrogen base/l and then pH-adjusted to 4.2 using concentrated HCl. Minimal inhibitory concentration (MIC) assays were conducted via broth microdilution in accordance with CLSI guidelines [21, 23] with the exception that test compounds were made up at 50x final assay concentration and 100 *μ*l assay volumes were used (2 *μ*l compound stock added to 98 *μ*l of broth containing cells at 0.5–2.5 × 10^3^ colony-forming units [CFU]/ml). MIC plates were read following a 24-h incubation at 35°C (or 48 h for some slower growing mutants) and MIC values are reported as the lowest concentrations resulting in prominent growth inhibition (~50%), as specified by CLSI for echinocandins and azoles or at complete inhibition for AMB [23]. Enumeration of MIC assay inoculum viable count was performed by plating 50 *μ*l of the starting assay inoculum on SDA. The full MIC panel was run three times, independently. MIC values for each drug/strain combination fell within 2-fold between replicates and modal MIC values are reported.

### 2.4. Quality Control

*C. krusei* ATCC 6258 was used as one of the representative* C. krusei* strains selected for this study and as a QC strain in susceptibility testing under standard CLSI conditions with AMB and FLU and for comparison to established CLSI QC ranges [21].

### 2.5. Time-Kill Assays

**Figure 1:**
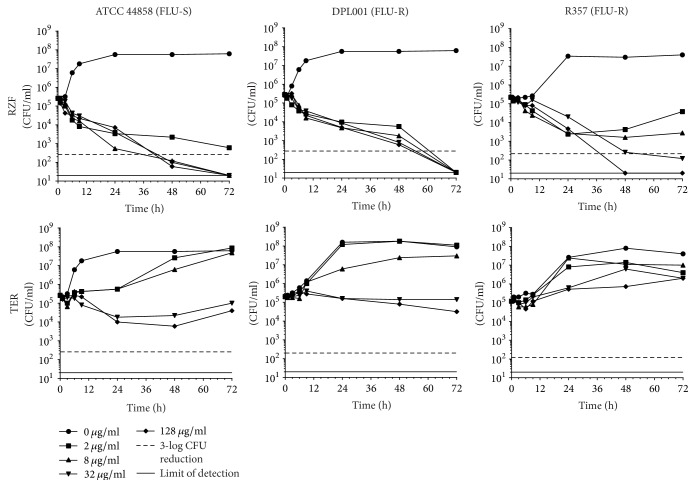
*C. albicans* time-kill curves for RZF and TER.

**Figure 2:**
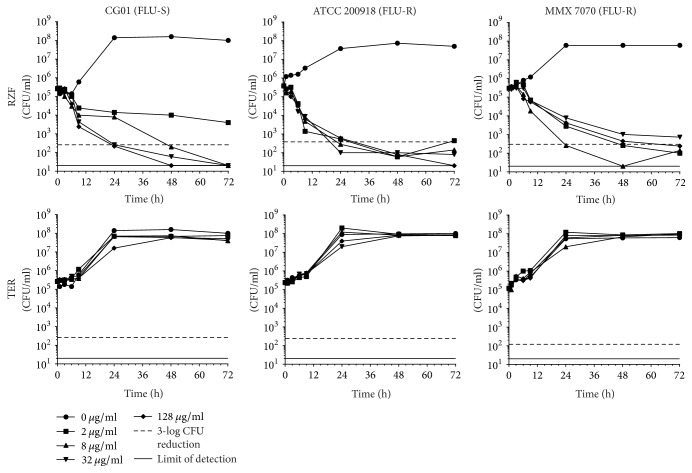
*C. glabrata* time-kill curves for RZF and TER.

**Figure 3:**
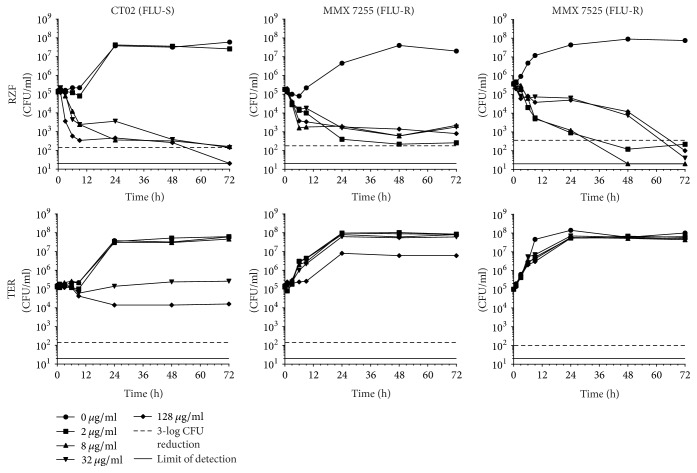
*C. tropicalis* time-kill curves for RZF and TER.

**Figure 4:**
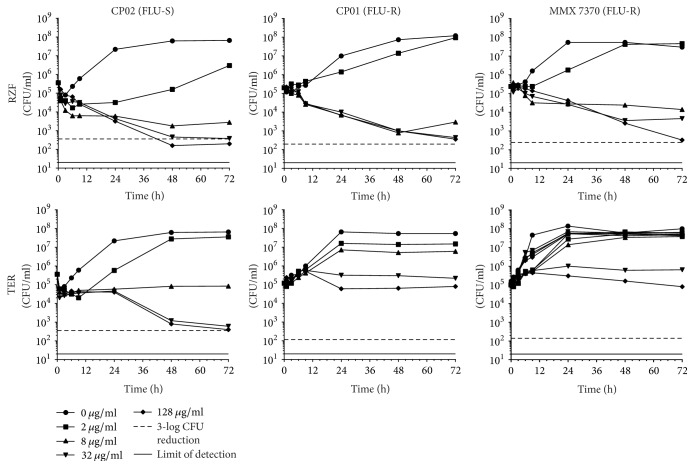
*C. parapsilosis* time-kill curves for RZF and TER.

**Figure 5:**
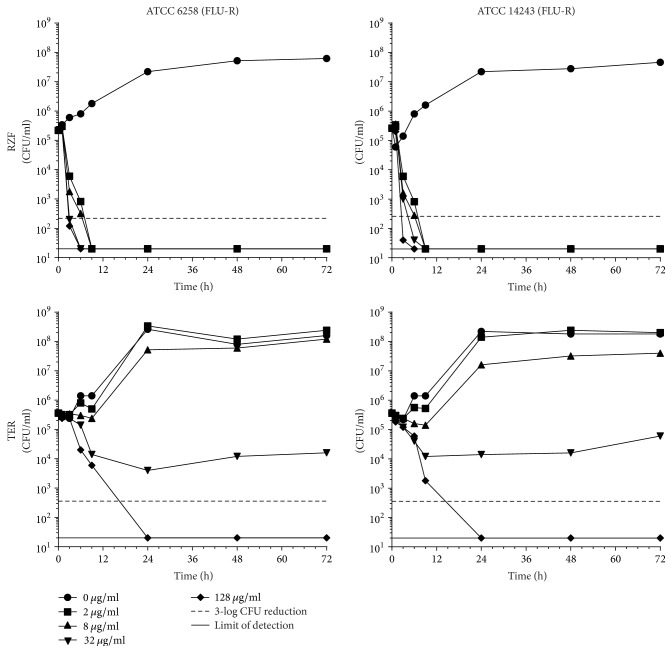
*C. krusei *time-kill curves for RZF and TER.

Time-kill assays were performed as previously described [24]. Overnight SDA cultures of each strain were resuspended in 0.85% NaCl to ~1.0 OD_530_ and added to 10 ml VSM to achieve an inoculum of 1.4 × 10^5^–3.8 × 10^5^ CFU/ml. Drugs were then added to the inoculated VSM to obtain concentrations of 0, 2, 8, 32, and 128 *μ*g/ml. Time-kills were performed in baffled, ventilated 125 ml Erlenmeyer flasks (TriForest, cat. number FBC0125S) incubated at 35°C in a shaking incubator. Aliquots were removed from each flask at 0, 1, 3, 6, 9, 24, 48, and 72 h and were pelleted/washed twice in 0.85% NaCl to remove residual drug and then serially diluted in 0.85% NaCl. Fifty microliters of each serial dilution was spread onto SDA plates and incubated for 24 h at 35°C (assay limit of detection: 20 CFU/ml; denoted by solid lines in Figures 1–5). Colonies were counted and CFU/ml values were calculated and plotted using GraphPad Prism 6 (GraphPad Software, Inc.). Fungistatic activity was defined by CFU reductions < 3-logs from the starting inoculum, and fungicidal activity was defined by CFU reductions ≥ 3-logs (denoted by dashed lines in Figures 1–5). Time-kills for each strain-drug combination were performed a single time.

## 3. Results

### 3.1. Susceptibility Testing

To establish baseline MIC values for RZF and azole comparators in VSM, CLSI broth microdilution values were generated for each* Candida* strain in both VSM at pH 4.2 and under standard CLSI test media and RPMI at pH 7.0 (Table 1). With the exception of* C. parapsilosis* (a* Candida* species with higher intrinsic echinocandin MIC values) RZF was 8- to >1,024-fold more potent than TER against all strains tested, in both media types (median 64-fold more potent). Under standard CLSI testing conditions in RPMI media, the 10 strains previously characterized as FLU-R by their respective sources maintained that phenotype, except for* C. glabrata* ATCC 200918 which came in one dilution under the “resistant” threshold, falling into the “susceptible-dose-dependent” designation [21]. In VSM media, RZF MIC values increased 2-fold (median) overall for RZF (range: 1- to 8-fold less potent), similarly to AMB. Between the azoles, median TER MIC values were 64-fold higher in VSM than for RPMI (range: 16-fold more potent to 512-fold less potent) while median FLU MIC values were ~3-fold less potent (range: >256-fold more potent to 16-fold more potent than in RPMI).

### 3.2. Quality Control

QC strains and susceptibility criteria are not available for MIC assays performed in VSM; however pH 7.0 RPMI MIC values derived for FLU and AMB for CLSI QC strain* C. krusei* ATCC 6258 fell within the established QC ranges (Table 1) [21].

### 3.3. Time-Kill Kinetics

A high-level summary of log-fold reductions in CFU for each strain/drug combination at 72 h is presented in Table 2 and individual time-kill curves are shown in Figures 1–5.

*(i) C. albicans*. RZF was fungicidal against all three* C. albicans *strains in a dose-dependent manner across the majority of concentrations tested (Figure 1). TER was fungistatic against all strains for which the largest effect observed versus the FLU-S strain ATCC 44858 was a ~1.6-log-fold decrease in CFU with the highest drug concentration tested (128 *μ*g/ml).

*(ii) C. glabrata*.* C. glabrata *strains were killed in a dose-dependent fungicidal manner by RZF across most concentrations tested, similarly to* C. albicans *(Figure 2). TER had limited inhibitory activity against all three strains with all concentrations tested resulting in ~3-logs of growth by 72 h, which was comparable to the no-drug control groups.

*(iii) C. tropicalis*. Fungicidal activity was observed for RZF versus FLU-S* C. tropicalis* CT02 by 72 h at 32 and 128 *μ*g/ml (Figure 3). Against FLU-R* C. tropicalis *strain MMX 7255, RZF was fungistatic at all concentrations tested (near-fungicidal for 128 *μ*g/ml and ~2-log-fold reductions for the other three concentrations). For FLU-R* C. tropicalis* strain MMX 7525, RZF was fungicidal by 72 h at all concentrations. At earlier time points (9, 24, and 48 h), the 2 and 8 *μ*g/ml groups generated greater log-fold-reductions in CFU than did the 32 and 128 *μ*g/ml groups. TER was fungistatic against all three* C. tropicalis* strains. The highest activity was observed at 32 and 128 *μ*g/ml versus the CT02 FLU-S strain, resulting in stasis or ~1-log CFU reductions, respectively. Little to no inhibitory activity was observed at the lower TER concentrations against this FLU-S strain or at any concentrations versus the two FLU-R strains.

*(iv) C. parapsilosis*. RZF was fungistatic against* C. parapsilosis *CP01 and MMX 7370 at all concentrations tested and was able to generate a ≥3-log CFU reduction against CP02 at 128 *μ*g/ml (Figure 4). Against all three strains, RZF exposure resulted in reductions in CFU by 72 h at all concentrations except 2 *μ*g/ml, where growth occurred. TER produced a similar pattern of killing profile over time to that of RZF versus CP02 but, unlike RZF, TER did not achieve 3-log cidality at any concentration including the highest tested (128 *μ*g/ml). For the two FLU-R* C. parapsilosis *strains, by 72 h, the 32 and 128 *μ*g/ml TER concentrations resulted in CFU stasis while growth was observed at 2 and 8 *μ*g/ml.

*(v) C. krusei*. RZF demonstrated rapid killing down to the assay limit of detection by 9 h against both strains of* C. krusei *and maintained these reductions through all remaining time points (Figure 5), at all concentrations. TER demonstrated fungicidal activity later, at 24 h, and only at the 128 *μ*g/ml concentration. At 32 *μ*g/ml, TER generated minor reductions in CFU or stasis, and growth was observed for both strains at 2 and 8 *μ*g/ml.

## 4. Discussion

This study demonstrated that RZF anti-*Candida* and fungicidal activity was retained under in vitro conditions relevant to topical, vaginal application through analysis in MIC assays and evaluation of time-kill kinetics. Furthermore, RZF demonstrated greater potency and fungicidal activity against a variety of* Candida* species than did TER.

The 5 species included in this study are representative of the most common* Candida* etiological pathogens of VVC [4, 8]. Where possible, clinical vaginal isolates were used (CG01, CP01, CP02, and CT02), although RZF MIC values derived herein and in prior MIC studies do not show a significant difference between strains of vaginal versus nonvaginal origin [20, 25]. In this study, MIC assays demonstrated that the anti-*Candida* activity of RZF under conditions simulating the vaginal environment (VSM pH 4.2) was minimally affected compared to its activity in media more closely mimicking systemic administration (RPMI pH 7.0). This retention of activity for RZF in VSM at pH 4.2 was similar to previous observations for RZF in RPMI media at pH 4.0 [20]. Some TER MIC values were lower in VSM than RPMI (*C. albicans*-only), but the majority were significantly higher in VSM and all TER MIC values in VSM were higher than the corresponding RZF MIC values in VSM. These data are consistent with analysis of azole agents versus VVC isolates across a range of pH values (4–7) in RPMI media [26, 27].

The test concentrations of RZF and TER of up to 128 *μ*g/ml in MIC and time-kill assays were consistent with the high local concentrations achievable by topical administration, although such drug concentrations presented a challenge and required modification of traditional time-kill methodology. Pilot time-kill studies revealed that use of these concentrations resulted in growth inhibition of surviving colonies due to residual drug carried over in the plating aliquots, so a wash step was incorporated into the protocol prior to CFU plating on agar media. Drug release rates of the RZF ointment and gel formulations prepared for clinical evaluation are 1% and 60%, respectively [28]. Administration of 4 ml of a 6% w/w ointment formulation (gel formulation: 3% w/w) at a drug release rate of 1% would deliver an intravaginal concentration of RZF in excess of 128 *μ*g/ml. On a more functional level, 128 *μ*g/ml also represents the solubility limit of RZF in RPMI media (unpublished observations). Although release rates and intravaginal concentration data for TER have not been published, these values for the approved 0.4 and 0.8% cream formulations would likely fall in between those of the RZF gel and ointment formulations with potentially lower intravaginal drug concentrations than RZF gel due to a lower formulation percentage.

In time-kill assays, RZF demonstrated fungicidal activity against 11 of 14* Candida* strains tested and was near-cidal against the remaining 3 strains. Of the five* Candida* spp. evaluated,* C. parapsilosis* exhibited the most similar killing kinetics between RZF and TER. The lower CFU reductions versus* C. parapsilosis* for RZF compared to the other* Candida* spp. in VSM are consistent with, although more pronounced than, trends observed at pH 7.0 in RPMI [18]. This killing profile is shared by other echinocandins against* C. parapsilosis* as well [29]. Also, the relatively increased killing of TER versus* C. parapsilosis* is consistent with enhanced azole activity against this species in time-kill experiments conducted under standard pH 7.0 conditions [30]. Despite higher MIC values and muted killing kinetics, echinocandins demonstrate strong efficacy against systemic infections caused by* C. parapsilosis* strains in vivo [31]. Whether a similar in vivo efficacy trend with topical RZF would be observed is yet to be determined.* C. parapsilosis* does comprise a relatively small percentage (typically < 5%) of all VVC isolates [4, 8], and the killing kinetics of RZF in VSM were fairly comparable to TER which is clinically efficacious as a topical treatment of VVC caused by* C. parapsilosis* [32].

RZF demonstrated the highest levels of killing versus* C. krusei* across all concentrations within 9 h in VSM, similar to killing kinetics observed at pH 7.0 in RPMI for* C. krusei* strains [18]. Echinocandins are fungicidal against all* Candida* species as compared to azoles which are fungistatic under standard testing conditions [33]. Yet, despite the characteristic fungistatic mechanism of action of the azole class, a study has shown that azoles can demonstrate fungicidal behavior against* C. albicans* under physiological vaginal conditions [22]. The incongruous in vivo efficacy of these agents in VVC is a phenomenon potentially explained by synergistic activity with acetic acid, as was demonstrated with FLU versus* C. albicans*,* C. parapsilosis*,* C. tropicalis*,* C. lusitaniae*, and* C. dubliniensis*, however not for* C. glabrata* or* C. krusei* [22]. Acetic acid is a component of the VSM used in this study and could be a contributing factor for instances where at least some fungicidal activity towards* C. krusei *was observed for TER at the highest concentration tested, despite this species being intrinsically resistant to azoles.

A similar incongruity has been observed for RZF, between the susceptibility and time-kill data presented herein as well as strong efficacy data from rat models of VVC [28], and the findings of RADIANT, a Phase 2 clinical trial of two topical formulations of RZF in patients with moderate to severe VVC. Clinical and mycological cures achieved with RZF validated the proof-of-concept of topical echinocandin treatment of acute VVC; however, neither formulation tested in the study generated efficacy data on par with oral fluconazole, and further development of topical RZF was discontinued [34]. Additional work elucidating potential efficacy-limiting factors (e.g., drug distribution, and drug release rates) would be needed to optimize formulations and/or dosing regimens for this indication. Future research may also include characterizing the activity of RZF against* Candida* biofilms, as recent studies have proposed that biofilms play a role in VVC [35], although their relevance is not entirely known [36]. Finally, the potential impact of native vaginal bacterial flora, such as lactobacilli, on the activity of RZF applied topically could be investigated to rule out any efficacy-hindering interactions.

## 5. Conclusions

An echinocandin therapeutic would introduce a novel mechanism of action for VVC, which has predominantly been treated with azole antifungals and remains an area of unmet medical need [2, 37]. The fungicidal activity could offer advantages over azoles in efficacy versus azole-S and azole-R isolates (in particular for* C. glabrata*). Furthermore, complete eradication of* Candida* through use of a cidal agent could improve or prevent the risk of recurrent disease. This study demonstrates the retention of anti-*Candida* activity and fungicidal killing kinetics for RZF under conditions relevant to VVC and at drug concentrations achievable through topical administration. These findings would support future efforts to optimize formulations and dosing strategies to enable translation of these desirable in vitro properties into in vivo efficacy for the treatment and prevention of VVC.

## Figures and Tables

**Table 1:** MIC values for time-kill assay *Candida* strains in VSM (pH 4.2) or RPMI (pH 7.0).

Species	Strain	FLU (S/R)^2^	MIC (*μ*g/ml)^1^							
			RZF		TER		FLU		AMB	
			VSM	RPMI	VSM	RPMI	VSM	RPMI	VSM	RPMI
*C. albicans*	ATCC 44858	S	0.06	0.03	2	32	1	1	1	0.125
	DPL001	R	0.03	0.06	64	32	128	>128	0.5	0.125
	R357	R	0.5	0.25	4	32	0.5	>128	0.5	0.25

*C. glabrata*	CG01	S	0.03	0.06	8	0.015	16	1	1	0.5
	ATCC 200918	R^3^	0.125	0.06	>128	1	>128	32	1	0.5
	MMX 7070	R	0.25	0.03	>128	2	>128	64	0.5	0.25

*C. tropicalis*	CT02	S	0.06	0.06	4	4	2	0.5	1	0.25
	MMX 7255	R	0.125	0.03	>128	64	>128	64	0.5	0.5
	MMX 7525	R	0.125	0.06	>128	4	>128	128	0.5	0.5

*C. parapsilosis*	CP02	S	2	2	4	0.03	2	0.25	1	0.25
	CP01	R	2	2	32	0.125	>128	16	1	0.25
	MMX 7370	R	2	1	32	0.5	64	64	1	0.5

*C. krusei*	ATCC 6258	R	0.125	0.06	16	0.5	64	32	1	0.5
	ATCC 14243	R	0.06	0.06	32	0.5	64	32	1	0.5

^1^MIC values were determined three times, independently. At most there was a 2-fold range in variation for each drug/strain combination MIC value between replicates and modal values are listed. ^2^Susceptibility to FLU as defined per CLSI 24 h broth microdilution interpretive criteria [21]. ^3^ATCC describes strain 200918 as FLU-R strain although the CLSI MIC value of 32 *μ*g/ml derived in this study is technically characterized as “susceptible-dose dependent” per CLSI interpretive criteria [21].

**Table 2:** Summary of time-kill log-fold changes in CFU at 72 h.

Species	Strain	Suscept. to FLU (S/R)	Drug	Log-fold change in CFU at each drug conc. (*μ*g/ml)			
				2	8	32	128
*C. albicans *	ATCC 44858	S	RZF	++	++++^*∗*^	++++^*∗*^	++++^*∗*^
			TER	-	-	+	+
	DPL001	R	RZF	++++^*∗*^	++++^*∗*^	++++^*∗*^	++++^*∗*^
			TER	-	-	-	-
	R357	R	RZF	+	++	+++^*∗*^	++++^*∗*^
			TER	-	-	-	-

*C. glabrata*	CG01	S	RZF	+	++++^*∗*^	++++^*∗*^	++++^*∗*^
			TER	-	-	-	-
	ATCC 200918	R	RZF	-^*∗*^	+++^*∗*^	+++^*∗*^	++++^*∗*^
			TER	-	-	-	-
	MMX 7070	R	RZF	+++^*∗*^	+++^*∗*^	++	+++^*∗*^
			TER	-	-	-	-

*C. tropicalis*	CT02	S	RZF	-	++	+++^*∗*^	+++^*∗*^
			TER	-	-	-	+
	MMX 7255	R	RZF	++	+	++	++
			TER	-	-	-	-
	MMX 7525	R	RZF	+++^*∗*^	++++^*∗*^	+++^*∗*^	+++^*∗*^
			TER	-	-	-	-

*C. parapsilosis*	CP02	S	RZF	+	++	++	+++^*∗*^
			TER	-	-	++	++
	CP01	R	RZF	-	++	++	++
			TER	-	-	-	-
	MMX 7370	R	RZF	-	+	+	++
			TER	-	-	-	-

*C. krusei*	ATCC 6258	R	RZF	++++^*∗*^	++++^*∗*^	++++^*∗*^	++++^*∗*^
			TER	-	-	+	++++^*∗*^
	ATCC 14243	R	RZF	++++^*∗*^	++++^*∗*^	++++^*∗*^	++++^*∗*^
			TER	-	-	-	++++^*∗*^

Symbols denote log-fold changes in CFU from starting inoculum: increase or <1-log reduction (-); ≥1- to <2-fold reduction (+); ≥2- to <3-fold reduction in CFU (++); ≥3- to <4-fold reduction in CFU (+++); ≥4- to <5-fold reduction in CFU (++++). *∗* indicates fungicidal activity (≥3-log CFU reduction).
